# Clinical Predictive Models for COVID-19: Systematic Study

**DOI:** 10.2196/21439

**Published:** 2020-10-06

**Authors:** Patrick Schwab, August DuMont Schütte, Benedikt Dietz, Stefan Bauer

**Affiliations:** 1 F Hoffmann-La Roche Ltd Basel Switzerland; 2 Eidgenössische Technische Hochschule Zürich Zürich Switzerland; 3 Max Planck Institute for Intelligent Systems Tübingen Germany

**Keywords:** SARS-CoV-2, COVID-19, machine learning, clinical prediction, prediction, infectious disease, clinical data, testing, hospitalization, intensive care

## Abstract

**Background:**

COVID-19 is a rapidly emerging respiratory disease caused by SARS-CoV-2. Due to the rapid human-to-human transmission of SARS-CoV-2, many health care systems are at risk of exceeding their health care capacities, in particular in terms of SARS-CoV-2 tests, hospital and intensive care unit (ICU) beds, and mechanical ventilators. Predictive algorithms could potentially ease the strain on health care systems by identifying those who are most likely to receive a positive SARS-CoV-2 test, be hospitalized, or admitted to the ICU.

**Objective:**

The aim of this study is to develop, study, and evaluate clinical predictive models that estimate, using machine learning and based on routinely collected clinical data, which patients are likely to receive a positive SARS-CoV-2 test or require hospitalization or intensive care.

**Methods:**

Using a systematic approach to model development and optimization, we trained and compared various types of machine learning models, including logistic regression, neural networks, support vector machines, random forests, and gradient boosting. To evaluate the developed models, we performed a retrospective evaluation on demographic, clinical, and blood analysis data from a cohort of 5644 patients. In addition, we determined which clinical features were predictive to what degree for each of the aforementioned clinical tasks using causal explanations.

**Results:**

Our experimental results indicate that our predictive models identified patients that test positive for SARS-CoV-2 a priori at a sensitivity of 75% (95% CI 67%-81%) and a specificity of 49% (95% CI 46%-51%), patients who are SARS-CoV-2 positive that require hospitalization with 0.92 area under the receiver operator characteristic curve (AUC; 95% CI 0.81-0.98), and patients who are SARS-CoV-2 positive that require critical care with 0.98 AUC (95% CI 0.95-1.00).

**Conclusions:**

Our results indicate that predictive models trained on routinely collected clinical data could be used to predict clinical pathways for COVID-19 and, therefore, help inform care and prioritize resources.

## Introduction

COVID-19 was first discovered in December 2019 in China and has since rapidly spread to over 200 countries [1]. The COVID-19 pandemic has challenged health care systems worldwide, as a high peak capacity for testing and hospitalization is necessary to diagnose and treat affected patients, particularly if the spread of SARS-CoV-2 is not mitigated. To avoid exceeding the available health care capacities, many countries have adopted social distancing policies, imposed travel restrictions, and postponed nonessential care and surgeries to reduce peak demand on their health care systems [2-4].

**Figure 1:**
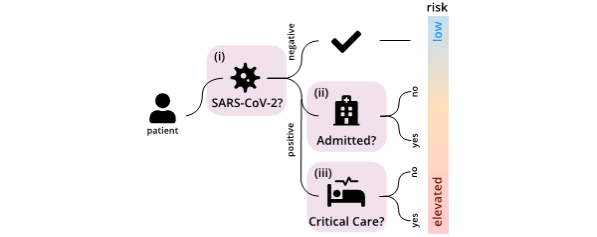
We study the use of predictive models (light purple) to estimate whether patients are likely (i) to be SARS-CoV-2 positive and whether SARS-CoV-2 positive patients are likely (ii) to be admitted to the hospital and (iii) to require critical care based on clinical, demographic, and blood analysis data. Accurate clinical predictive models stratify patients according to individual risk and, in this manner, help prioritize health care resources such as testing, hospital, and critical care capacity.

The adoption of clinical predictive models that accurately predict who is likely to require testing, hospitalization, and intensive care from routinely collected clinical data could potentially further reduce peak demand by ensuring resources are prioritized to those individuals with the highest risk (Figure 1). For example, a clinical predictive model that accurately identifies patients that are likely to test positive for SARS-CoV-2 a priori could help prioritize limited SARS-CoV-2 testing capacity. However, developing accurate clinical prediction models for SARS-CoV-2 is difficult as relationships between clinical data, hospitalization, and intensive care unit (ICU) admission have not yet been established conclusively due to the recent emergence of SARS-CoV-2.

In this systematic study, we develop and evaluate clinical predictive models that use routinely collected clinical data to identify patients that are likely to receive a positive SARS-CoV-2 test, patients who are SARS-CoV-2 positive that are likely to require hospitalization, and patients who are SARS-CoV-2 positive that are likely to require intensive care. Using the developed predictive models, we additionally determined which clinical features are most predictive for each of the aforementioned clinical tasks. Our results indicate that predictive models could be used to predict clinical pathways for patients with COVID-19. Such predictive models may be of significant utility for health care systems, as preserving health care capacity has been linked to successfully combating SARS-CoV-2 [5,6].

Concretely, this paper contains the following contributions:

- We developed and systematically studied predictive models for estimating the likelihoods of a positive SARS-CoV-2 test in patients presenting at hospitals, hospital admission in patients who are SARS-CoV-2 positive, and critical care admission in patients who are SARS-CoV-2 positive.
- We validated the performance of the developed clinical predictive models in a retrospective evaluation using real-world data from a cohort of 5644 patients.
- We determined and quantified the predictive power of routinely collected clinical, demographic, and blood analysis data for the aforementioned clinical prediction tasks.

## Methods

### Problem Setting

In the present setting, we are given 106 routine clinical, laboratory, and demographic measurements, or features, *x_i_* ∈ *x* for presenting patients (see Multimedia Appendix 1 for full list). Features may be discrete or continuous, and some features may be missing as not all tests are necessarily performed on all patients. The clinical predictive tasks consist of using the routine clinical features *x_i_* to predict, for a newly presenting patient, the likelihood *ŷ_SARS-CoV-2_* of receiving a positive SARS-CoV-2 test result, the likelihood *ŷ_admission_* of requiring hospital admission, and the likelihood *ŷ_ICU_* of requiring intensive care. In addition, we are given a development data set consisting of *N* patients, their corresponding observed routine clinical features *x_i_*, SARS-CoV-2 test results *ŷ_SARS-CoV-2_* ∈ {0,1}, hospital admissions *ŷ_admission_* ∈ {0,1}, and ICU admissions *ŷ_ICU_* ∈ {0,1}, where 1 indicates the presence of an outcome. Using this development data set, our goal is to derive clinical predictive models 
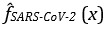
, 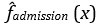, and 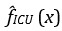 for the aforementioned tasks, respectively, to inform care and help prioritize scarce health care resources.

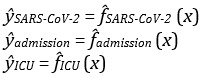



### Methodology

**Figure 2:**
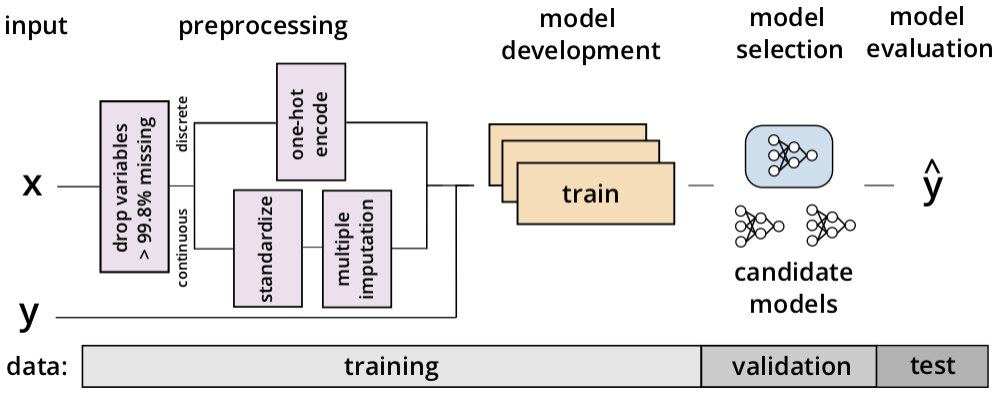
The presented multistage machine learning pipeline consists of preprocessing (light purple) the input data x, developing multiple candidate models using the given data set (orange), selecting the best candidate model for evaluation (blue), and evaluating the selected best model's outputs ŷ.

To derive the clinical predictive models 
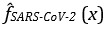
, 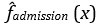, and 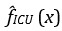 from the given development data set, we set up a systematic model development, validation, and evaluation pipeline (Figure 2). To evaluate the generalization ability of the developed clinical predictive models and to rule out overfitting to patients in the evaluation cohort, the development data is initially split into independent and stratified training, validation, and test folds without any patient overlap. Concretely, the multistage pipeline consists of preprocessing, model development, model selection, and model evaluation stages. For preprocessing and model development, only the training fold was used, and only the validation and test folds of the development data were used for model selection and model evaluation, respectively. We outline the pipeline stages in detail in the following paragraphs.

### Preprocessing

In the preprocessing stage, we first dropped all input features that were missing for more than 99.8% of all training set patients to ensure we had a minimal amount of data for each feature. This removed a total of 9 features from the original 106 routine clinical, laboratory, and demographic features. We then transformed all discrete features for each patient into their one-hot encoded representation with one out of *p* indicator variables set to 1 to indicate the discrete value for this patient, and all others set to 0 with *p* being the number of unique values for the discrete feature. We defined those features as discrete that have fewer than 6 unique values across all patients in the training fold. For discrete features, missing features were counted as a separate category in the one-hot representation. Next, we standardized all continuous features to have zero mean and unit standard deviation across the training fold data. Last, we performed multiple imputation by chained equations (MICE) to impute all missing values of every continuous feature from the respective other features in an iterative fashion [7]. We additionally added a missing indicator that indicates 1 if the feature was imputed by MICE and 0 if it was originally present to preserve missingness information in the data after imputation. After the preprocessing stage, continuous input features are standardized and fully imputed, and discrete input features are one-hot encoded. All preprocessing operations were derived only from the training fold and naïvely applied without adjustment to validation and test folds to avoid information leakage.

### Model Development

In the model development stage, we trained candidate clinical predictive models 
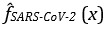
, 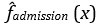, and 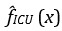 using supervised learning on the training fold of the preprocessed data. To derive the models from the preprocessed training fold data, we optimized various types of predictive models and performed a hyperparameter search with *m* runs for each of them. The model development process yielded *m* candidate models with different hyperparameter choices and predictive performances for each model category.

### Model Selection

To select the best model among the set of candidate models, we evaluated their predictive performance against the held-out validation fold that had not been used for model development. We chose the top candidate model by ranking all models by their evaluated predictive performance in terms of the validation set area under the receiver operator characteristic curve (AUC). The model selection stage using the independent validation fold enabled us to optimize hyperparameters without using test fold data.

### Model Evaluation

In the model evaluation stage, we evaluated the selected best clinical predictive model against the held-out test fold that had not been used for training or model selection to estimate the expected generalization error of the models on previously unseen data. Using this approach, every selected best model from the model selection stage was evaluated exactly once against the test fold.

Using the presented standardized model development, selection, and evaluation pipeline, we compared various types of clinical predictive models in the same test setting with exactly the same amount of hyperparameter optimization and input features against the same test fold. This process enables us to systematically study the expected generalization ability, predictive performance, and influential features of clinical predictive models for predicting SARS-CoV-2 test results, hospital admission for patients who are SARS-CoV-2 positive, and ICU admission for patients who are SARS-CoV-2 positive.

### Experiments

We conducted retrospective experiments to evaluate the predictive performance of a number of clinical predictive models on each of the presented clinical prediction tasks using the standardized development, validation, and evaluation pipeline.

Concretely, our experiments aimed to answer the following questions:

- What is the expected predictive performance of the various clinical predictive models in predicting SARS-CoV-2 test results for presenting patients, hospital admission for patients who are SARS-CoV-2 positive, and ICU admission for patients who are SARS-CoV-2 positive?
- Which clinical, demographic, and blood analysis features were most important for the best encountered predictive models for each clinical prediction task?

### Data Set and Study Cohort

We used anonymized data from a cohort of 5644 patients seen at the Hospital Israelita Albert Einstein in São Paulo, Brazil in the early months of 2020. Exact data collection dates are unknown. The data set is available at [8]. Over the data collection time frame, the rate of patients who were SARS-CoV-2 positive at the hospital was around 10%, of which around 6.5% and 2.5% required hospitalization and critical care, respectively (Table 1). Notably, younger patients were underrepresented in the SARS-CoV-2 positive group relative to the general patient population, which may have been caused by the reportedly more severe disease progression in older patients [9]. Information on patient sex was not included in our data set. Sex has been reported to be associated with COVID-19 outcomes with men reportedly being at higher risk for severe outcomes, and models including sex as a covariate may, therefore, achieve superior predictive performance [10]. We randomly split the entire available patient cohort into training (n=2822, 50%), validation (n=1129, 20%), and test folds (n=1693, 30%) within strata of patient age, SARS-CoV-2 test result, hospital admission status, and ICU admission status. We performed the stratification by randomly shuffling the entire set of available patients and then assigning a proportional number of patients within the same strata of patient age, SARS-CoV-2 test result, hospital admission status, and ICU admission status to each fold, resulting in three separate folds of the desired target fold sizes that had balanced proportions of the stratification covariates (Table 1). We used the implementation of the described stratification procedure provided in [11] (StratifiedShuffleSplit, package version 0.22.2).

**Table 1:** Training, validation, and test fold statistics for all patients and patients who are SARS-CoV-2 positive.

Property		Training	Validation	Test
**All patients**				
	Patients (N=5644), n (%)	2822 (50)	1129 (20)	1693 (30)
	SARS-CoV-2 (%)	9.85	9.92	9.92
	Admission (%)	1.42	1.33	1.42
	ICU^a^ (%)	1.59	1.68	1.59
	Age (20-quantiles)^b^	9.0 (1.0, 17.0)	9.0 (1.0, 18.0)	9.0 (2.0, 17.0)
**Patients who are SARS-CoV-2 positive**				
	Patients (n=558), n (%)	279 (50)	112 (20)	167 (30)
	SARS-CoV-2 (%)	100	100	100
	Admission (%)	6.45	6.25	6.59
	ICU (%)	2.87	2.68	2.99
	Age (20-quantiles)^b^	10.0 (4.0, 17.0)	11.5 (4.5, 18.5)	10.0 (4.0, 17.5)

^a^ICU: intensive care unit.

^b^Patient ages are specified in 20-quantiles to maintain patient privacy (10% and 90% percentiles in parentheses).

### Models

Using the presented systematic evaluation methodology, we trained five different model types: logistic regression (LR), neural network (NN), random forest (RF), support vector machine (SVM), and gradient boosting (XGB) [12]. The NN was a multilayer perceptron consisting of *L* hidden layers with *N* hidden units each followed by a nonlinear activation function (rectified linear unit [13], scaled exponential linear unit [14], or exponential linear unit [15]) and batch normalization [16], and was trained using the Adam optimizer [17] for up to 300 epochs with an early stopping patience of 12 epochs on the validation set loss.

### Hyperparameters

We followed an unbiased, systematic approach to hyperparameter selection and optimization. For each type of clinical predictive model, we performed a maximum of 30 hyperparameter optimization runs with hyperparameters chosen from predefined ranges (Table 2). The performance of each hyperparameter optimization run was evaluated against the validation cohort. After computing the validation set performance, we selected the best candidate predictive model across the 30 hyperparameter optimization runs by AUC for further evaluation against the test set.

**Table 2:** Hyperparameter ranges used for hyperparameter optimization of logistic regression, neural network, random forest, support vector machine, and gradient boosting models for all tasks.

Model and hyperparameter		Range/choices^a^
**Logistic regression**		
	Regularization strength *C*	0.01, 0.1, 1.0, 10.0
**Neural network**		
	Number of hidden units *N*	16, 32, 64, 128
	Number of hidden layers *L*	1, 2, 3
	Activation *a*	ReLU^b^ [13], SELU^c^ [14], ELU^d^ [15]
	Batch size *B*	16, 32, 64, 128
	L2 regularization *λ*_2_	0.0, 0.00001, 0.0001
	Learning rate *α*	0.003, 0.03
	Dropout percentage *p*	(0%-25%)
**Random forest**		
	Tree depth D	3, 4, 5
	Number of Trees T	32, 64, 128, 256
**Support vector machine**		
	Regularization strength C	0.01, 0.1, 1.0, 10.0
	Kernel k	polynomial, radial basis function, sigmoid
	Polynomial degree d	3, 5, 7
**Gradient boosting**		
	Subsample ratio r	0.25, 0.5, 0.75, 1.0
	Max^e^ tree depth T	2, 3, 4, 5, 6, 7, 8
	Min^f^ partition loss *γ*	0.0, 0.1, 1.0, 10.0
	Learning rate *α*	0.003, 0.03, 0.3, 0.5
	L1 regularization *λ*_1_	1.0, 0.1, 0.001, 0.0
	L2 regularization *λ*_2_	1.0, 0.1, 0.001, 0.0
	Num^g^ boosting rounds B	5, 10, 15, 20

^a^Parentheses indicate continuous ranges within the indicated limits sampled uniformly. Comma-delimited lists indicate discrete choices with equal selection probability.

^b^ReLU: rectified linear unit.

^c^SELU: scaled exponential linear unit.

^d^ELU: exponential linear unit.

^e^Max: maximum.

^f^Min: minimum.

^g^Num: number.

### Predictive Performance

To assess the predictive performance of each of the developed clinical predictive models, we evaluated their performance in terms AUC, area under the precision recall curve (AUPR), sensitivity, specificity, and specificity at greater than 95% sensitivity (Spec@95%Sens) on the held-out test set cohorts for each task (Table 1). After model development and hyperparameter optimization, we evaluated each model type exactly once against the test set to calculate the final performance metrics. Operating thresholds for each model were the operating points on the receiver operator characteristic curve closest to the top left coordinate as calculated for the validation cohort. We chose a variety of complementary evaluation metrics to give a comprehensive picture of the expected performance of each clinical predictive model on the evaluated tasks. For each of the performance metrics, we additionally computed 95% CIs using bootstrap resampling with 100 bootstrap samples on the test set cohort to quantify the uncertainty of our analysis results. We also assessed whether differences between clinical predictive models were statistically significant at significance level α=.05 using pairwise *t* tests with the respective best models for each task as measured by AUC.

### Importance of Test Types

To quantify the importance of specific clinical, demographic, and blood analysis features on each of the predicted outcomes, we used causal explanation (CXPlain) models [18]. CXPlain provides standardized relative feature importance attributions for any predictive model by computing the marginal contribution of each input feature toward the predictive performance of a model [19] and is, therefore, particularly well-suited for assessing feature importance in our diverse set of models. We used the test fold’s ground truth labels to compute the exact marginal contribution of each input feature without any estimation uncertainty.

## Results

### Predictive Performance

In terms of predictive performance (Table 3), we found that the overall best identified models by AUC were XGB for predicting SARS-CoV-2 test results, RF for predicting hospital admissions for patients who are SARS-CoV-2 positive, and SVM for predicting ICU admission for patients who are SARS-CoV-2 positive with AUCs of 0.66 (95% CI 0.63-0.70), 0.92 (95% CI 0.81-0.98), and 0.98 (95% CI 0.95-1.00), respectively. Notably, we found that predicting positive SARS-CoV-2 results from routinely collected clinical measurements was a considerably more difficult task for clinical predictive models than predicting hospitalization and ICU admission. Nonetheless, the best encountered clinical predictive model for predicting SARS-CoV-2 test results (XGB) achieved a respectable sensitivity of 75% (95% CI 67%-81%) and specificity of 49% (95% CI 46%-51%). After fixing the operating threshold of the model to meet a sensitivity level of at least 95% (Spec@95%Sens), the best XGB model for predicting SARS-CoV-2 test results would achieve a specificity of 23% (95% CI 7%-32%). We additionally found that the differences in predictive performance between the best XGB model for predicting SARS-CoV-2 test results and the other predictive models was significant at a prespecified significance level of α=.05 (*t* test) for all but the AUPR metric, where NN achieved a significantly better AUPR of 0.22, and the difference to SVM was not significant at the prespecified significance level. On the task of predicting hospital admissions for patients who are SARS-CoV-2 positive, the best encountered RF model achieved a sensitivity of 55% (95% CI 19%-85%), a high specificity of 96% (95% CI 92%-98%), and a Spec@95%Sens of 34% (95% CI 29%-97%). Owing to the lower sample size due to the smaller cohort of patients who are SARS-CoV-2 positive, the performance results for predicting hospital admission generally had wider uncertainty bounds but were nonetheless significantly better for RF than the other predictive models at the prespecified significance level of α=.05 (*t* test) for most performance metrics, with the exception of AUC, where XGB achieved an AUC of 0.91, and AUPR, where LR achieved an AUPR of 0.44. On the task of predicting ICU admission for patients who are SARS-CoV-2 positive, SVM had a sensitivity of 80% (95% CI 36%-100%), a specificity of 96% (95% CI 92%-98%), and a Spec@95% Sens of 95% (95% CI 91%-100%). Due to the small percentage of about 3% of patients who were SARS-CoV-2 positive that were admitted to the ICU (Table 1), uncertainty bounds were wider than for the models predicting hospital admissions, and the results of the best encountered SVM were found to be not significantly better than LR and RF in terms of AUC, LR, and NN in terms of sensitivity, and NN in terms of Spec@95%Sens at the prespecified significance level of α=.05 (*t* test).

**Table 3:** Comparison of LR, NN, RF, SVM, and XGB models in terms of AUC, AUPR, sensitivity, specificity, and Spec@95%Sens for predicting SARS-CoV-2 test results, hospital admission for patients who are SARS-CoV-2 positive, and intensive care unit admission for patients who are SARS-CoV-2 positive on the test set cohort.

Model		AUC^a^ (95% CI)^b^		AUPR^c^ (95% CI)		Sensitivity (95% CI)		Specificity (95% CI)		Spec@95%Sens^d^ (95% CI)	
**SARS-CoV-2 test results**											
	XGB^e^		*0.66*^f^ (0.63-0.70)		0.21 (0.15-0.28)		*0.75* (0.67-0.81)		0.49 (0.46-0.51)		*0.23* (0.07-0.32)
	RF^g^		0.65 (0.62-0.69)^h^		0.19 (0.14-0.24)^h^		0.69 (0.61-0.74)		0.54 (0.46-0.57)^h^		0.19 (0.10-0.25)^h^
	NN^i^		0.62 (0.57-0.65)^h^		*0.22* (0.15-0.28)^h^		0.60 (0.52-0.67)^h^		0.55 (0.46-0.58)^h^		0.17 (0.14-0.28)^h^
	LR^j^		0.61 (0.57-0.65)^h^		0.17 (0.13-0.24)^h^		0.58 (0.51-0.65)^h^		0.55 (0.46-0.57)^h^		0.19 (0.16-0.25)^h^
	SVM^k^		0.61 (0.57-0.65)^h^		0.21 (0.15-0.27)		0.57 (0.51-0.64)^h^		*0.59* (0.56-0.61)^h^		0.14 (0.06-0.16)^h^
**Hospital admissions for patients who are SARS-CoV-2 positive**											
	RF		*0.92* (0.81-0.98)		0.43 (0.19-0.81)		0.55 (0.19-0.85)		*0.96* (0.92-0.98)		*0.34* (0.29-0.97)
	XGB		0.91 (0.80-0.98)		*0.52* (0.28-0.84)^h^		0.64 (0.43-0.95)^h^		0.94 (0.90-0.97)^h^		0.00 (0.00-0.94)^h^
	LR		0.88 (0.70-0.98)^h^		0.44 (0.18-0.83)		*0.82* (0.52-1.00)^h^		0.85 (0.79-0.90)^h^		0.13 (0.08-0.93)^h^
	NN		0.85 (0.68-0.97)^h^		0.31 (0.13-0.66)^h^		0.64 (0.33-1.00)^h^		0.95 (0.91-0.97)^h^		0.11 (0.06-0.93)^h^
	SVM		0.85 (0.70-0.98)^h^		0.35 (0.17-0.77)^h^		0.64 (0.30-1.00)^h^		0.95 (0.91-0.97)^h^		0.21 (0.15-0.96)^h^
**Critical care admissions for patients who are SARS-CoV-2 positive**											
	SVM		*0.98* (0.95-1.00)		0.53 (0.14-1.00)		*0.80* (0.36-1.00)		0.96 (0.92-0.98)		*0.95* (0.91-1.00)
	LR		*0.98* (0.93-1.00)		*0.67* (0.09-1.00)^h^		*0.80* (0.29-1.00)		0.93 (0.89-0.96)		0.91 (0.87-1.00)^h^
	NN		0.97 (0.94-0.99)^h^		0.35 (0.10-0.88)^h^		*0.80* (0.36-1.00)		0.95 (0.91-0.99)^h^		0.94 (0.90-0.99)
	RF		0.97 (0.92-1.00)		0.56 (0.13-1.00)^h^		0.60 (0.15-1.00)^h^		*0.98* (0.96-1.00)^h^		0.90 (0.86-1.00)^h^
	XGB		0.67 (0.53-0.98)^h^		0.29 (0.01-0.68)^h^		0.40 (0.00-1.00)^h^		0.94 (0.91-0.97)^h^		0.00 (0.00-0.96)^h^

^a^AUC: area under the receiver operator characteristic curve.

^b^95% CIs obtained via bootstrap resampling with 100 samples.

^c^AUPR: area under the precision recall curve.

^d^Spec@95%Sens: specificity at greater than 95% sensitivity.

^e^XGB: gradient boosting.

^f^Italics represent the best results.

^g^RF: random forest.

^h^Significant at *P*<.05 (*t* test) to the model with the highest predictive performance in terms of AUC.

^i^NN: neural network.

^j^LR: logistic regression.

^k^SVM: support vector machine.

### Feature Importance

**Figure 3:**
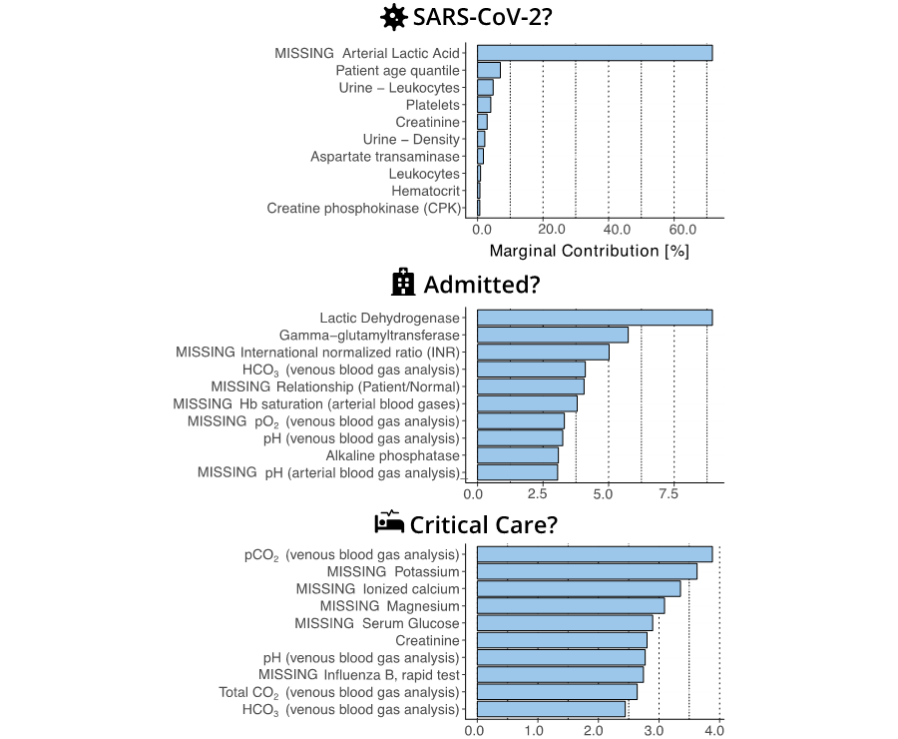
A comparison of the top 10 features ranked by relative feature importance scores for the best-encountered model for predicting SARS-CoV-2 test results (gradient boosting, top), hospital admissions (random forest, middle), and critical care admission for patients who are SARS-CoV-2 positive (support vector machine, bottom), respectively. The bar length corresponds to the relative marginal importance (in %) of the displayed features toward the predictive performance of the respective model. Feature names that include “MISSING” indicate that the given marginal contribution refers to the importance of the presence of that feature's absence, not the feature itself.

In terms of feature importance, we found that importance scores were distributed highly unequally, relatively uniform, and highly uniform for the best models encountered for predicting SARS-CoV-2 test results, for predicting hospital admissions for patients who are SARS-CoV-2 positive, and for predicting ICU admission, respectively (Figure 3). Most notably, we found that 71.7% of the importance for the best XGB model for predicting SARS-CoV-2 test results was assigned to the missing indicator corresponding to the arterial lactic acid measurement (ie, much of the marginal predictive performance gain of the XGB model was attributed to whether or not the arterial lactic acid test had been ordered). Beyond arterial lactic acid being missing, age, leukocyte count, platelet count [20], and creatinine [21] were implied to be associated with a positive SARS-CoV-2 test result by the best encountered predictive model, which further substantiates recent independent reports of those factors being potentially associated with SARS-CoV-2 [20-24]. Similar to the best encountered XGB model for predicting SARS-CoV-2 test results, the top encountered predictive models for hospital admission and ICU admission for patients who are SARS-CoV-2 positive assigned a considerable degree of importance to missingness patterns associated with a number of measurements. A possible explanation for missingness appearing as a top predictor across the different tasks is that decisions on whether or not to order a certain test to be performed for a given patient were influenced by patient characteristics that were not captured in the set of clinical measurements that were available to the predictive models. In the case of the missingness of the lactic acid test being predictive of SARS-CoV-2 test results, the importance could stem either from clinicians judging patients to be more likely to have COVID-19 due to their clinical presentation and, therefore, ordering a lactic acid test to account for potential lactic acidosis due to COVID-19–induced reduced oxygenation levels or from patients that clinicians see as at risk for lactic acidosis being likely to have their symptoms caused by an underlying SARS-CoV-2 infection. A controlled setting with standardized testing guidelines would be required to determine which confounding factors are behind the predictive power of the missingness patterns that have been implied to be associated with COVID-19 by the predictive models. Beyond missingness patterns, top predictors for predicting hospital admission were lactate dehydrogenase [25]; gamma-glutamyl transferase, which through abnormal liver function has been reported to be implicated in COVID-19 severity [26]; and *HCO*_3_ [27]. For predicting ICU admission in patients who are SARS-CoV-2 positive, *pCO*_2_, creatinine [21], and pH [23] were top predictors. Blood pH, and in particular respiratory alkalosis, has been reported to be associated with severe COVID-19 [28]. We note that several factors that were not included in our study have recently been reported to be implicated in COVID-19 outcomes, such as the number of ICU beds available at a hospital [29], patients’ racial and ethnic backgrounds [30], and several pre-existing conditions [31].

## Discussion

### Principal Findings

**Figure 4:**
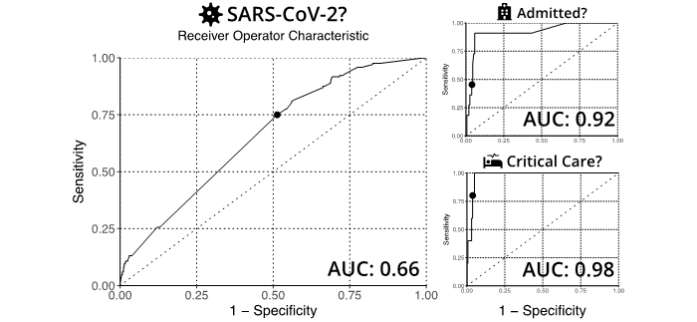
Receiver operator characteristic curves for the best-encountered model for predicting SARS-CoV-2 test results (gradient boosting, left), hospital admissions for patients who are SARS-CoV-2 positive (random forest, top right), and critical care admissions for patients who are SARS-CoV-2 positive (support vector machine, bottom right). Numbers in the bottom right of each subgraph show the respective model's AUC. Solid dots on the curves indicate operating thresholds selected on the validation fold. AUC: Area under the receiver operator characteristic curve.

We presented a systematic study of predictive models that predict SARS-CoV-2 test results, hospital admission for patients who are SARS-CoV-2 positive, and ICU admission for patients who are SARS-CoV-2 positive using routinely collected clinical measurements. Models that predict SARS-CoV-2 test results could help prioritize scarce testing capacity by identifying those individuals that are more likely to receive a positive result. Similarly, predictive models that predict which patient who is SARS-CoV-2 positive would be most likely to require hospital and critical care beds could help better use existing hospital capacity by prioritizing those patients that have the highest risk of deterioration. Facilitating the efficient use of scarce health care resources is particularly important in dealing with SARS-CoV-2, as its rapid transmission significantly increases demand for health care services worldwide (Figure 4).

### Limitations

The main limitation of this study is that its experimental evaluation was based on data collected from a single study site, and its results may, therefore, not generalize to settings with significantly different patient populations, admission criteria, patterns of missingness, and testing guidelines. Operationally, to ensure robustness, it is important to check for any significant deviations in terms of patterns observed in the training cohort when attempting to transfer predictive models trained in one context to another (ie, when transferring a model to another hospital). In case any significant deviations are detected, fine-tuning the predictive model for the new target context is strongly advised. In addition, we did not have access to mortality data for the analyzed cohort, and we were, therefore, not able to correlate our predicted individual risk scores with patient mortality, which is another related prediction task that may be of clinical importance. Future studies should include a broader set of clinical measurements and outcomes, cohorts from multiple distinct geographical sites, and under varying patterns of missingness to determine the robustness of the clinical predictive models to these confounding factors. Finally, we believe that the inclusion of data from other modalities such as genomic profiling and medical imaging, and data on comorbidities, symptoms, and treatment histories could potentially further improve predictive performance of clinical predictive models across the presented prediction tasks.

### Comparison With Prior Work

A substantial body of work is dedicated to the study, validation, and implementation of predictive models for clinical tasks. Clinical predictive models have, for example, been used to predict risk of septic shock [32,33], risk of heart failure [34], readmission following heart failure [35-37], false alarms in critical care [38], risk scores [39], outcomes [40] and mortality in pneumonia [41,42] , and mortality risk in critical care [43-45]. Predicting clinical outcomes for individual patients is difficult because many confounding factors may influence patient outcomes, and collecting and accounting for these factors in an unbiased way remains an open challenge in clinical practice [46]. Systematic studies such as this paper enable medical practitioners to better understand, assess, and potentially overcome these issues by systematically evaluating generalization ability, expected predictive performance, and influential predictors of various clinical predictive models. Beyond the need for systematic evaluation, missingness [47-50], noise [51,52], multivariate input data [38,53-55], and the need for interpretability [18,56-58] have been highlighted as particularly important considerations in health care settings. In this study, we build on recent methodological advances to develop and systematically study clinical predictive models that may aid in prioritizing health care resources [59] for COVID-19 and, thereby, help prevent a potential overextension of health care system capacity.

### Clinical Predictive Models for COVID-19

Several clinical predictive models have recently been proposed for COVID-19, for example, for predicting potential COVID-19 diagnoses using data from emergency care admission exams [60] and chest imaging data [61-66], for predicting COVID-19–related mortality from clinical risk factors [67,68], for predicting which patients will develop acute respiratory distress syndrome from patients’ clinical characteristics [69], for predicting critical illness in patients with COVID-19 [70,71], and for predicting progression risk in patients with COVID-19 pneumonia [72]. Siordia [73] presented a review of epidemiology and clinical features associated with COVID-19, and Wynants et al [74] performed a critical review that assessed limitations and risk of bias in diagnostic and prognostic models for COVID-19. In addition, Wang et al [23] performed a cohort study for clinical and laboratory predictors of COVID-19–related in-hospital mortality that identified baseline neutrophil count, age, and several other clinical features as top predictors of mortality. Beyond prediction, Ienca and Vayena [75] have argued for the responsible use of data in tackling the challenges posed by SARS-CoV-2.

Owing to the recent emergence of SARS-CoV-2, there currently exists, to the best of our knowledge, no prior systematic study on clinical predictive models that predict the likelihood of a positive SARS-CoV-2 test and hospital and ICU admission from clinical, demographic, and blood analysis data that accounts for the missingness that is characteristic for the clinical setting. We additionally assessed the influence of various clinical, demographic, and blood analysis measurements on the predictions of the developed clinical predictive models.

### Conclusions

We present a systematic study in which we developed and evaluated clinical predictive models for COVID-19 that estimate the likelihood of a positive SARS-CoV-2 test in patients presenting at hospitals and the likelihood of hospital admission and ICU admission in patients who are SARS-CoV-2 positive. We evaluated our developed clinical predictive models in a retrospective evaluation using a cohort of 5644 hospital patients seen in São Paulo, Brazil. In addition, we determined the clinical, demographic, and blood analysis measurements that were most important for accurately predicting SARS-CoV-2 status, hospital admissions, and ICU admissions. Our experimental results indicate that clinical predictive models may in the future potentially be used to inform care and help prioritize scarce health care resources by assigning personalized risk scores for individual patients using routinely collected clinical, demographic, and blood analysis data. Furthermore, our findings on the importance of routine clinical measurements toward predicting clinical pathways for patients increases our understanding of the interrelations of individual risk profiles and outcomes in SARS-CoV-2. Based on our study’s results, we conclude that health care systems should explore the use of predictive models that assess individual COVID-19 risk to improve health care resource prioritization and inform patient care.

## Appendix

Multimedia Appendix 1 Demographic, clinical, and blood analysis data used as features by our systematic model development and evaluation pipeline, and their respective value ranges in the data set.

## Abbreviations

AUC: area under the receiver operator characteristic curve
AUPR: area under the precision recall curve
CXPlain: causal explanation
ICU: intensive care unit
LR: logistic regression
MICE: multiple imputation by chained equations
NN: neural network
RF: random forest
Spec@95%Sens: specificity at greater than 95% sensitivity
SVM: support vector machine
XGB: gradient boosting
