# Supplementary material for: Clinical Predictive Models for COVID-19: Systematic Study
Source: J Med Internet Res. 2020 Oct 6;22(10):e21439. doi: 10.2196/21439 (PMC7541040; doi:10.2196/21439)
Supplement: Multimedia Appendix 1 [file jmir_v22i10e21439_app1.docx]

## Multimedia Appendix 1

Table 4. Demographic, clinical and blood analysis data used as features by our systematic model development and evaluation pipeline, and their respective value ranges in the utilized dataset. All values were standardized prior to data release for privacy reasons, and the observed value ranges prior to standardization are therefore not known to the authors.

| Name | Value range |
| --- | --- |
| Patient age quantile | 20-quantiles |
| Haematocrit | standardised scalar |
| Haemoglobin | standardised scalar |
| Platelets | standardised scalar |
| Mean platelet volume | standardised scalar |
| Red blood cells | standardised scalar |
| Lymphocytes | standardised scalar |
| Mean corpuscular haemoglobin concentration (MCHC) | standardised scalar |
| Leukocytes | standardised scalar |
| Basophils | standardised scalar |
| Neutrophils | standardised scalar |
| Eosinophils | standardised scalar |
| Mean corpuscular haemoglobin (MCH) | standardised scalar |
| Mean corpuscular volume (MCV) | standardised scalar |
| Monocytes | standardised scalar |
| Red blood cell distribution width (RDW) | standardised scalar |
| Serum glucose | standardised scalar |
| Respiratory syncytial virus | missing, detected, not detected |
| Influenza A | missing, detected, not detected |
| Influenza B | missing, detected, not detected |
| Influenza A H1N1 2009 | missing, detected, not detected |
| Influenza A, rapid test | missing, detected, not detected |
| Influenza B, rapid test | missing, detected, not detected |
| Strepto A | missing, detected, not detected |
| Parainfluenza 1 | missing, detected, not detected |
| Parainfluenza 2 | missing, detected, not detected |
| Parainfluenza 3 | missing, detected, not detected |
| Parainfluenza 4 | missing, detected, not detected |
| Rhinovirus / Enterovirus | missing, detected, not detected |
| Mycoplasma pneumoniae | missing, detected, not detected |
| Chlamydophila pneumoniae | missing, detected, not detected |
| Coronavirus HKU1 | missing, detected, not detected |
| Coronavirus NL63 | missing, detected, not detected |
| Coronavirus 229E | missing, detected, not detected |
| Coronavirus OC43 | missing, detected, not detected |
| Adenovirus | missing, detected, not detected |
| Bordetella pertussis | missing, detected, not detected |
| Metapneumovirus | missing, detected, not detected |
| Urea | standardised scalar |
| C-reactive Protein | standardised scalar |
| Creatinine | standardised scalar |
| Potassium | standardised scalar |
| Sodium | standardised scalar |
| Magnesium | standardised scalar |
| Phosphor | standardised scalar |
| Alanine transaminase | standardised scalar |
| Aspartate transaminase | standardised scalar |
| Gamma-glutamyltransferase | standardised scalar |
| Total Bilirubin | standardised scalar |
| Direct Bilirubin | standardised scalar |
| Indirect Bilirubin | standardised scalar |
| Alkaline phospatase | standardised scalar |
| Ionized calcium | standardised scalar |
| pCO_2_ (venous blood gas analysis) | standardised scalar |
| pO_2_ (venous blood gas analysis) | standardised scalar |
| Hb saturation (venous blood gas analysis) | standardised scalar |
| Base excess (venous blood gas analysis) | standardised scalar |
| FiO_2_ (venous blood gas analysis) | standardised scalar |
| Total CO_2_ (venous blood gas analysis) | standardised scalar |
| pH (venous blood gas analysis) | standardised scalar |
| HCO_3_ (venous blood gas analysis) | standardised scalar |
| pCO_2_ (arterial blood gas analysis) | standardised scalar |
| pO_2_ (arterial blood gas analysis) | standardised scalar |
| Hb saturation (arterial blood gas analysis) | standardised scalar |
| Base excess (arterial blood gas analysis) | standardised scalar |
| FiO_2_ (arterial blood gas analysis) | standardised scalar |
| Total CO_2_ (arterial blood gas analysis) | standardised scalar |
| pH (arterial blood gas analysis) | standardised scalar |
| HCO_3_ (arterial blood gas analysis) | standardised scalar |
| ctO_2_ (arterial blood gas analysis) | standardised scalar |
| Rods # | standardised scalar |
| Segmented | standardised scalar |
| Promyelocytes | standardised scalar |
| Metamyelocytes | standardised scalar |
| Myelocytes | standardised scalar |
| Myeloblasts | standardised scalar |
| Esterase (urine) | missing, not detected |
| Aspect (urine) | missing, clear, lightly cloudy, cloudy |
| pH (urine) | standardised scalar |
| Haemoglobin (urine) | missing, not detected, detected |
| Bile pigments (urine) | missing, not detected |
| Ketone bodies (urine) | missing, not detected |
| Nitrite (urine) | standardised scalar |
| Density (urine) | standardised scalar |
| Urobilinogen (urine) | missing, normal |
| Protein (urine) | missing, not detected |
| Sugar (urine) | standardised scalar |
| Leukocytes (urine) | standardised scalar |
| Crystals (urine) | missing, not detected, calcium oxalate -++, calcium oxalate +++ |
| Red blood cells (urine) | standardised scalar |
| Hyaline cylinders (urine) | missing, not detected |
| Granular cylinders (urine) | missing, not detected |
| Yeasts (urine) | missing, not detected |
| Colour (urine) | missing, yellow, citrus yellow, light yellow |
| Partial thromboplastin time (PTT) | standardised scalar |
| Relationship (Patient/Normal) | standardised scalar |
| International normalized ratio (INR) | standardised scalar |
| Lactic dehydrogenase | standardised scalar |
| Prothrombin time (PT), Activity | standardised scalar |
| Vitamin B12 | standardised scalar |
| Creatine phosphokinase (CPK) | standardised scalar |
| Ferritin | standardised scalar |
| Arterial lactic acid | standardised scalar |
| Lipase dosage | standardised scalar |
| D-dimer | standardised scalar |
| Albumin | standardised scalar |
